# Smartphone-Assisted Thin-Layer Chromatography for Rapid Quality Screening of Metformin

**DOI:** 10.1155/adpp/3306550

**Published:** 2025-06-18

**Authors:** Ram Kumar Bhattarai, Sanam Pudasaini, Toni Barstis, Basant Giri

**Affiliations:** ^1^Center for Analytical Sciences, Kathmandu Institute of Applied Sciences, Kathmandu, Nepal; ^2^Department of Chemistry and Physics, Saint Mary's College, Notre Dame, Indiana, USA

**Keywords:** diabetic, drug quality, low-quality drugs, metformin, smartphone application, *TLC Analyzer*

## Abstract

Type II diabetes remains a significant global public health issue, affecting both individual well-being and healthcare systems worldwide. Metformin hydrochloride is widely prescribed as the first-line treatment for managing diabetes. However, the increasing reports of substandard and falsified medicines, including metformin, circulating in the markets in recent years, highlights the urgent need for reliable and portable quality assurance tools. Thin-layer chromatography (TLC) has long been extensively used as a screening method to verify the identity and quality of various medicines. In this work, we present a smartphone-assisted TLC method for quantitative analysis of metformin hydrochloride. The TLC was performed using silica gel 60 F_254_ plates as the stationary phase and acetic acid-methanol-water 0.25:7:4 (v/v) as a mobile phase. We used a custom-made UV-illuminated TLC imaging box to capture images via smartphone and images were analyzed using a custom written smartphone application to calculate the *R*_*f*_ and the concentration of metformin in the principal TLC spots. The smartphone application, *TLC Analyzer*, accurately calculated the *R*_*f*_ values (0.604) consistent with those obtained using ImageJ software. The linearity of the method was 0.5–4 mg/mL. After optimization, the *TLC Analyzer* method was used to analyze metformin samples (*n* = 16) collected from local pharmacies. The results were compared with those from ImageJ analysis, UV–Vis spectrophotometry, and HPLC. The smartphone-based *TLC Analyzer* method identified 15 of the 16 samples as containing acceptable levels of metformin in accordance with pharmacopeial standards, consistent with ImageJ and spectrophotometric results. In contrast, the HPLC method indicated that all 16 samples met the pharmacopeial criteria.

## 1. Introduction

Type II diabetes is considered a serious public health concern with considerable impact on human life and healthcare expenditure [1], and its prevalence is rising across various regions globally. Approximately 415 million people live with diabetes worldwide, with Type II diabetes accounting for over 90% of the patients [2]. Metformin hydrochloride is widely used as the first line of medication for Type II diabetes and functions as an oral antihyperglycemic agent to lower blood sugar levels in affected patients [3]. Beyond its primary use, metformin has shown its potential therapeutic benefits in other diseases. Emerging evidences suggest a decrease in the incidence of cardiovascular diseases among patients with Type II diabetes [4], while observational data in humans also suggest its possible role in delaying aging and preventing cancer [4, 5]. In recent years, reports of substandard and falsified metformin products have risen across various countries, including Japan [6], Nepal [7], India, Nigeria, and Cambodia [8]. These studies showed that the metformin samples failed in key quality assessment parameters, including doses, uniformity testing, and dissolution testing.

The widespread circulation of low-quality medicines is a barrier to providing quality health care worldwide [9]. The low-quality medicines include substandard, falsified, unregistered, and unlicensed medical products. Substandard medicines, also known as out-of-specification drugs, are authorized medical products that fail to meet quality standards or specifications. Falsified medicines, on the other hand, are intentionally and fraudulently misrepresented in terms of their identity, composition, or source. Additionally, the unregistered or unlicensed medical products are those not approved by the relevant regulatory authorities [10, 11]. This issue of poor-quality medical products is particularly prevalent in developing countries. A recent meta-analysis showed, on average, 13.6% of medicines in low- and middle-income countries are of low quality, with the prevalence reaching up to 18.7% in Africa, resulting in an estimated economic impact of $10 billion to $200 billion [12]. The consequences of substandard and falsified medical products are wide-ranging, including elevated disease prevalence, loss of public trust in health systems, rising mortality and morbidity, economic losses, wastage resources, and broader socioeconomic impacts such as reduced productivity, income loss, limited social mobility, and increased poverty [10]. For instance, a 10% prevalence of poor-quality antibiotics could result in 72,000 to 169,00 childhood pneumonia deaths, while 2.1%–4.9% of malaria deaths in sub-Saharan Africa are attributed to such substandard and falsified medicines [10]. These alarming figures highlight the urgent need for cost-effective, efficient, and accessible methods to test the quality of medicines, particularly in point-of-care settings.

Several analytical methodologies have been developed to assess the quality of drugs, including metformin. These methods include high-performance liquid chromatography (HPLC), liquid chromatographic-tandem mass spectrometry, capillary electrophoresis, and UV-visible spectroscopy [13]. While these well-established methods offer high accuracy and reliability, they are often time-consuming, expensive, and reliant on highly trained personnel, which limit their routine use for quality testing of metformin and other drugs in question, particularly in resource-constrained countries.

Thin-layer chromatography (TLC) offers a simple, rapid, and low-cost approach for initial screening of pharmaceutical products like metformin [14]. Its affordability and ease of use make TLC especially promising for resource-limited settings. Several field deployable TLC kits have been developed for this purpose, including the Speedy TLC Kit [14], Global Pharma Health Fund (GPHF)'s Minilab [15], and Field Forensic Inc.‘s micro-TLC [16]. These kits typically work by comparing the TLC profile of an unknown sample with that of a known reference standard. However, many of these systems rely on expensive imaging devices and computers for analyzing TLC images, which limits their practicality in resource limited settings [17]. Smartphones offer a promising alternative, presenting an affordable and portable solution for image capture and analysis. They have already been applied in a variety of field-level analytical tasks, including biomarker detection, fluorescence sensing, and paper-strip interpretation [17–21].

In this study, we designed and developed a custom-built UV imaging box and an Android software application for TLC image analysis (named as *TLC Analyzer*) aimed at assessing drug quality. Using metformin as a model pharmaceutical product, the Android *TLC Analyzer* incorporated an advanced image processing algorithm to classify each pixel of the TLC image as either a spot or background, enabling automatic calculation of retention factors (*R*_*f*_) and accurate quantification of spot color intensity for quantitative analysis. To assess the reliability of the new method, we evaluated its analytical performance with the widely used ImageJ software. Furthermore, we validated the accuracy of our approach by analyzing metformin samples from local markets and comparing the results with those obtained from UV spectrophotometry and HPLC. Our results demonstrate the potential of low-cost and portable solutions for reliable and accessible drug quality screening.

## 2. Experimental Methods

### 2.1. Chemicals and Reagents

Acetic acid, ethanol, methanol, sodium hydroxide, sodium nitroprusside (SNP), and sodium hypochlorite (NaOCl) were purchased from Thermo Fisher Scientific India Pvt. Ltd., India. The metformin standard was bought from Accord Healthcare Pvt. Ltd., India. Precoated silica gel 60 F_254_ TLC plates were obtained from Merck, Germany. All the chemicals used were of analytical grade and were used as obtained.

### 2.2. Sample Collection and Sample Preparation

A standard stock solution of metformin was prepared by dissolving 100 mg in 10 mL of 50% ethanol. Working standard solutions with concentrations ranging from 0.5 to 4 mg/mL were then prepared by serial dilution of the stock solution using ethanol.

To validate the *TLC Analyzer* method described in this study, 16 metformin samples were obtained from local pharmacies by secret student shoppers, ensuring that the pharmacies were unaware the products would undergo quality testing. For each sample, 10 metformin tablets (500 mg) were weighed to calculate the average tablet weight. The tablets were then wrapped in aluminum foil and ground into a fine powder using a pestle. A quantity of powder equivalent to one tablet was dissolved in 25 mL of 50% ethanol, allowed to stand overnight, and subsequently stirred for at least 1 hour. The resulting sample solution was filtered, and the filtrate was diluted with ethanol to prepare a working solution with a final concentration of 4 mg/mL.

### 2.3. TLC

**Figure 1:**
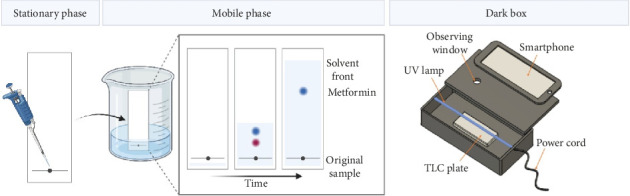
General procedure of TLC. A known concentration of metformin standard and unknown samples are spotted onto the plate (left panel). The TLC plate is developed in a chamber containing the mobile phase to elute the samples (central panel). The image of the plate, once dried, is taken in a dark box (right panel).

Chromatographic analysis was performed using 20 × 20 cm precoated silica gel 60 F254 TLC plates (Merck, Germany). The mobile phase consisted of acetic acid-methanol-water in a ratio of 0.25:7:4 (v/v). Linear ascending development was carried out in a 50 mL glass beaker serving as the developing chamber. Prior to development, the chamber was presaturated with the mobile phase up to 2 cm height of plate for 15 min at room temperature. A 4 μL volume of both standard and metformin sample solutions was loaded onto the TLC plates. Once the mobile phase migrated to 1 cm from the top edge, the plates were removed, air-dried, and imaged under UV excitation in the imaging box. A general overview of the TLC procedure is depicted in Figure 1.

A box measuring 25 cm × 15 cm × 15 cm was constructed from 0.5 mm thick cardboard sheets (Figure 1). A 2 cm rectangular hole was created in the lid of the box to allow image capture using the rear camera of a smartphone. The TLC plate was inserted into the box through a front-facing entrance slit. Since the metformin principal spots are not visible to the naked eye, UV excitation at 237 nm was used to visualize them. Under UV light, the metformin absorbs the green light emitted by the fluorescent compound in the plate, resulting in dark spots where the drug is present.

### 2.4. Image Processing Algorithm

Each TLC image was analyzed using a custom-made Android application named *TLC Analyzer,* built using Android Studio [22]. We incorporated OpenCV library V3.42 [23] for image capture and processing. The application allows the user to capture images in red-green-blue (RGB) format or load RGB images from the phone's gallery. After loading the image, the application enabled cropping to the required region, specifically between two solvent fronts. The cropped image was split to extract the green channel, followed by inversion and normalization using the min-max algorithm implemented in OpenCV. A 2D Gaussian filter (5 × 5 kernel, sigma = 0) was applied to smooth the image and reduce high-frequency noise. Subsequent image dilation was performed using a 16 × 16 kernel, after which binary thresholding was applied. The resulting image was a binary matrix, where pixels equal to or above the threshold value were set to 1, and those below were set to 0.

The *TLC Analyzer* then utilized OpenCV's contour detection function to identify the contours within the binary image. The moment function was used to detect the center points of each detected contours. Once the contours were identified, a bounding rectangle was drawn around each contour to isolate the corresponding spot. These contours were then mapped onto the original green channel image, and the area under the curve (AUC) within each contour was calculated to quantify the intensity of each spot, enabling accurate assessment of metformin concentration.

To analyze a sample TLC profile, the *TLC Analyzer* captured and processed the image, then displayed the concentration and retention factor (*R*_*f*_) of the spots on the TLC plate. The *R*_*f*_ for each spot was calculated as the ratio of the distance traveled by the compound to the distance traveled by the solvent front, both measured from the origin line.

(1) $$\begin{array}{c} R_{f}=\frac{\text{Distance travelled by the prinicipal spot}}{\text{Distance travelled by the solvent front}}. \end{array}$$

**Figure 2:**
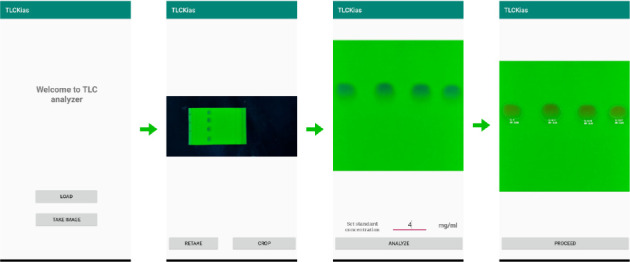
Screenshots demonstrating the *TLC Analyzer* workflow.

The summary of the app's working mechanism is shown in Figure 2. At first, the user can either capture a new image of the TLC plate or load a previously saved image from the smartphone. Once the image is loaded onto the app, the region containing the principal spots is selected for analysis. If the image captured is out of focus or suffers from inconsistent lighting, the user has the option to retake the image and repeat the process to ensure accurate results.

To compare the performance of the *TLC Analyzer*, the same images were also analyzed using ImageJ software [24]. Each image was split into its red, green and blue channels, with the green channel selected for quantification. Both the *TLC Analyzer* and ImageJ calculated the AUC for each spot on the TLC plate. Drug concentration was then determined using a calibration curve generated from standard metformin solutions. The source code for the *TLC Analyzer* has been made publicly available on GitHub at https://github.com/Sanam597/TLCKiasV2.

### 2.5. Limit of Detection (LOD) and Limit of Quantification (LoQ)

The LoD and LoQ were calculated using the equations: LoD = 3.3∗(Sy/S) and LoQ = 10∗ (Sy/S), where Sy is the standard deviation of the calibration curve and S is its slope. All experiments were performed in triplicate, and the values are reported as the mean ± standard deviation.

We conducted repeatability and recovery experiments to verify the precision of the method.

### 2.6. Spectrophotometric and HPLC Measurements

To further confirm and validate the results obtained from the *TLC Analyzer*, 16 pharmaceutical samples were quantitatively analyzed using an LVS-A20 UV-visible spectrophotometer (LABTRON, U.K.) and a standard HPLC method. Calibration curves were generated using the reference standard in the range of 5–50 μg/mL. A tablet-equivalent weight of each sample was dissolved in 10 mL of water and subsequently diluted to get the final drug concentration of 30 μg/mL. The spectrophotometric signal was measured at 236 nm [25].

HPLC measurements were carried out at Saint Mary's College using Waters Alliance e2695 system equipped with column heater and autosampler. The HPLC setup consisted of a 2487 UV/Vis detector and Luna 5 um C18(2) 100A, LC Column 250 × 4.6 mm (S/N H21-090486; B/N: 5291-0202) with SecurityGuard Cartridges (C18 4 × 3.0 mm ID, AJO-4290). Both samples and standards were prepared in Nanopure Water. The mobile phase consisted of a 20:80 (v/v) mixture of acetonitrile and buffer solution. The buffer solution consisted of 0.5 g/L of sodium octanesulfonate and 0.5 g/L of NaCl in water, maintained to a pH of 3.85. A 5.0 μL sample was injected at a flow rate of 1.0 mL/min, with a total run time of 7 min. The method demonstrated a precision of 0.44% RSD (10 uL of 0.5 mg/mL metformin, *n* = 6) and an average recovery of 104% for 500 mg metformin. Representative chromatograms of a standard and a sample metformin are provided in supporting information (Figures S1, S2).

## 3. Results and Discussion

### 3.1. Performance of Android *TLC Analyzer*

#### 3.1.1. Repeatability

**Figure 3:**
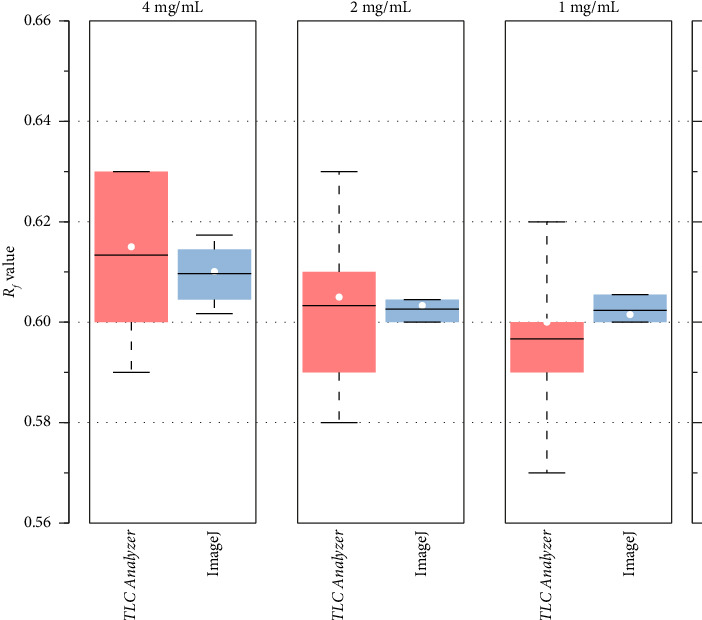
Box plots showing a comparison of *R*_*f*_ values with *TLC Analyzer* and ImageJ methods.

We evaluated the repeatability of the Android-based *TLC Analyzer* by measuring the *R*_*f*_ values of standard metformin at concentrations ranging from 1 to 4 mg/mL (Figure 3). The average *R*_*f*_ value obtained using the *TLC Analyzer* was 0.604 ± 0.017 (*n* = 18). Although *R*_*f*_ values showed a slight increase with the increase in the concentration, the difference was not statistically significant (*p* > 0.05). For comparison, the average *R*_*f*_ value calculated using the ImageJ method was 0.605 ± 0.005 (*n* = 18), closely matching the results obtained from the *TLC Analyzer*.

The repeatability of the methods was evaluated by measuring the coefficient of variance (CV) of the *R*_*f*_ values, defined as the ratio of the standard deviation (*σ*) to the mean (μ).

(2) $$\begin{array}{c} \text{CV}=\frac{\sigma }{µ}. \end{array}$$

Using this formula, the average CV calculated with the *TLC Analyzer* was 2.8% (*n* = 18), while the ImageJ method yielded a lower average CV of 0.9%. These results indicate that the *TLC Analyzer* exhibited higher variability in *R*_*f*_ measurements compared to the ImageJ software run on a laptop (Table 1).

**Figure 4:**
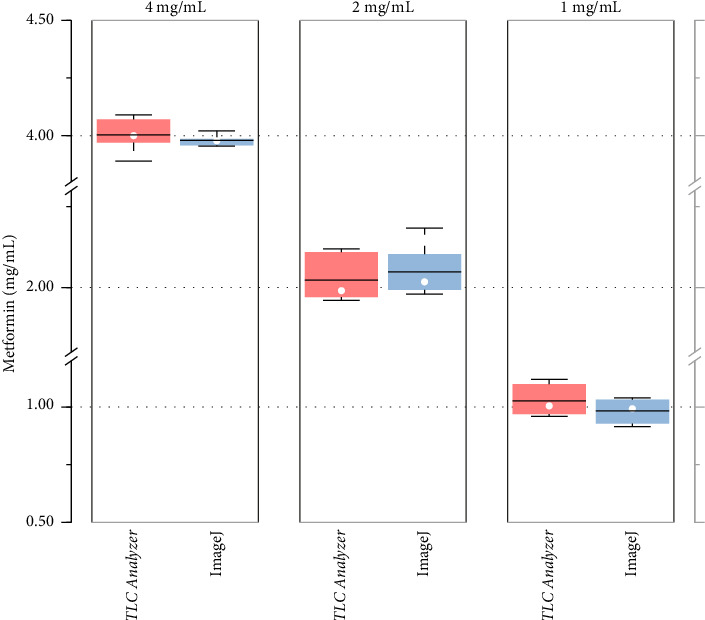
Comparison of *TLC Analyzer* and ImageJ methods to predict the concentration of metformin. Experiments were conducted for 4 mg/mL (left), 3 mg/mL (center), and 1 mg/mL (right) metformin, each with six independent trials.

The repeatability of the *TLC Analyzer* method was further demonstrated by calculating the concentrations of standard metformin as shown in Figure 4. Each concentration—4 mg/mL, 2 mg/mL, and 1 mg/mL—was measured independently six times. The average concentrations (*n* = 6) measured by the *TLC Analyzer* were found to be 4.00 ± 0.07 mg/mL, 2.02 ± 0.07 mg/mL and 1.03 ± 0.07 mg/mL, respectively. The corresponding CVs were 1.8%, 3.6%, and 6.7%. Similarly, the average concentrations (*n* = 6) using the ImageJ method were 3.98 ± 0.02 mg/mL, 2.05 ± 0.07 mg/mL and 0.98 ± 0.05 mg/mL for the same standard concentrations, with respective CV of 0.5%, 3.5%, and 4.8%. Both methods demonstrated comparable accuracy and repeatability in measuring metformin concentrations. Notably, the CV decreased as the concentration of metformin increased in both methods (Table S1).

#### 3.1.2. Recovery Experiments

Standard metformin solutions ranging from 1.0 mg/L to 4.0 mg/mL were spiked and analyzed using the TLC. The resulting TLC plates were analyzed by both the *TLC Analyzer* and ImageJ to calculate the recovery rates. The percentage recovery for the *TLC Analyzer* ranged from 96.61 ± 3.71% to 102.67 ± 6.93%, while the ImageJ method yielded recoveries between 98.37 ± 4.76% to 102.44 ± 3.62% (Table 2). No significant differences in recovery were observed across the various metformin concentrations for either method.

#### 3.1.3. Calibration Curve

**Figure 5:**
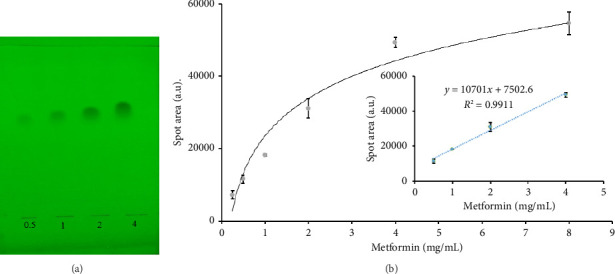
(a) Sample TLC plates with different concentrations of metformin and (b) calibration curve relating the peak area of TLC plate of metformin to its concentration in mg/mL.

We created a response curve with metformin standard solutions ranging from 0.25 to 8 mg/mL using the ImageJ method (Figure 5). The response was linear in the range of 0.5–4 mg/mL range, with *R*^2^ > 0.99. The LoD and LoQ were found to be 1.45 and 4.41 mg/mL, respectively. The regression equation for the linear range, *Y* = 107,01*X* + 7502, was used to estimate the concentrations of metformin in pharmaceutical samples. A different equation (*Y* = 2404.70*X* + 9006.9) was fed into the *TLC Analyzer* algorithm as images were cropped in different aspect ratio with the app.

### 3.2. Validation of the *TLC Analyzer* Method Using Metformin Tablet Samples

After optimizing the *TLC Analyzer* method, we applied it to determine the active pharmaceutical ingredient (API) content in metformin tablets purchased from local pharmacies in Kathmandu (*n* = 16). The samples were analyzed using the *TLC Analyzer* following the same method for standards described in Section 2.3. As outlined in the methodology Section 2.4, the *TLC Analyzer* measured two key parameters of each sample: the *R*_*f*_ value and spot intensity. The *R*_*f*_ value of each sample was compared to that of the standard to confirm the presence of metformin, while the spot intensity was applied to the regression equation to quantify the API content in each tablet sample.

According to the Indian Pharmacopeia, a drug sample is considered genuine if its API content is within ±10% of the labeled claim. For 500 mg metformin tablets, this translates to an acceptable range of 450–550 mg per tablet. Samples were considered substandard if their API content fell outside this range or if their *R*_*f*_ values deviated by more than 10% from the standard. The analysis results for the sample are presented in Table 3.

The *TLC Analyzer* resulted in an average *R*_*f*_ value of 0.60 ± 0.02 across all samples, with all 16 samples falling within 10% of the standard metformin *R*_*f*_ value. Similarly, the ImageJ method yielded an average *R*_*f*_ value of 0.61 ± 0.01, closely aligning with the *TLC Analyzer* results (Table 3). Based on this *R*_*f*_ comparison, the *TLC Analyzer* correctly identified all tested samples as containing metformin API.

The *TLC Analyzer* quantified API content in all 16 samples and found 15 of them were within the acceptable range of 500 ± 50 mg/tablet, as specified by the Indian Pharmacopeia. One sample, MT38, was below the threshold, with an API content of 413.75/tablet. To evaluate the performance of the *TLC Analyzer*, we also tested the same set of 16 samples using ImageJ, UV–vis spectrophotometer, and HPLC. The results from both the ImageJ and spectrophotometric methods were consistent with the *TLC Analyzer*, identifying 15 samples within the acceptable limit and one (MT38) below it.

An ANOVA test showed no statistically significant difference between the results of the *TLC Analyzer* and the ImageJ method (*p* = 0.301), or between the *TLC Analyzer* and the spectrophotometric analysis (*p* = 0.675). In contrast, HPLC analysis showed that all tested samples, including MT38, were within the acceptable range, with MT38 registering 479.02 mg/tablet (see Supporting Information). Nonetheless, the difference between the HPLC and *TLC Analyzer* results for MT38 was not statistically significant (*p* = 0.587).

## 4. Conclusion

This study presents a novel smartphone-assisted TLC method for rapid and cost-effective screening of metformin quality. The *TLC Analyzer*, combined with a custom-built UV imaging box, successfully detected and quantified metformin in pharmaceutical samples. It demonstrated high accuracy in retention factor determination, closely matching results obtained using the widely adopted ImageJ software. Quantitative analysis showed that 15 out of 16 samples tested were within the acceptable API content range (500 ± 50 mg/tablet) as per Indian Pharmacopeia standards, with one substandard sample (MT38) identified across the *TLC Analyzer*, ImageJ, and UV–vis spectrophotometric methods. ANOVA testing confirmed no statistically significant differences among these methods (*p* > 0.05), and while HPLC results found all samples within the acceptable range—including MT38 at 479.02 mg/tablet—the difference from *TLC Analyzer* readings was not statistically significant (*p* = 0.587). These findings validate the reliability and accuracy of the smartphone-based TLC approach. By offering an accessible, affordable, and portable alternative to conventional laboratory equipment, the *TLC Analyzer* method fills a critical gap in pharmaceutical quality control, especially in resource-limited settings. Its demonstrated ability to identify substandard products using simple smartphone platform underscores its practical potential. Future developments should focus on improving automation, integrating AI for result interpretation, and expanding the drug database to broaden its application in global medicine quality surveillance and strengthen efforts against substandard and falsified pharmaceuticals.

## Figures and Tables

**Table 1:** Comparison of CV values for measuring *R*_*f*_ using *TLC Analyzer* and ImageJ software.

Method used	% CV of *R*_*f*_ measurement			
	4 mg/mL	2 mg/mL	1 mg/mL	Average
*TLC Analyzer*	2.7	2.9	2.7	2.8
ImageJ	1.0	0.3	0.4	0.9

**Table 2:** Relative recoveries received for samples.

Spiked (mg/mL)	*TLC Analyzer* result		ImageJ result	
	mg/mL	Relative recovery (%)	mg/mL	Relative recovery (%)
4.0	4.00 ± 0.07	100.08 ± 1.83	3.98 ± 0.02	99.48 ± 0.55
3.6	3.60 ± 0.10	100.16 ± 2.96	3.54 ± 0.08	98.56 ± 2.26
3.0	2.89 ± 0.12	96.61 ± 3.71	3.00 ± 0.07	100.13 ± 2.45
2.4	2.39 ± 0.07	99.85 ± 0.40	2.37 ± 0.04	98.75 ± 1.81
2.0	2.02 ± 0.07	101.17 ± 3.66	2.05 ± 0.07	102.44 ± 3.62
1.0	1.03 ± 0.07	102.67 ± 6.93	0.98 ± 0.05	98.37 ± 4.76

**Table 3:** Comparison of metformin concentration in the samples using TLC/ImageJ, *TLC Analyzer*, spectrophotometer, and HPLC.

Sample ID	*R*_*f*_ value		Metformin API content (mg/tablet)			
	*TLC Analyzer*	ImageJ	*TLC Analyzer*	ImageJ	UV-visible spectrometer	HPLC
MT30	0.62	0.61	468.75	487.59	500.74	473.64
MT38	0.60	0.60	*413.75*	*403.01*	*419.08*	479.02
MT33	0.59	0.61	495.00	493.14	489.42	473.93
MT12	0.61	0.59	486.25	461.82	498.07	481.59
MT82	0.62	0.60	500.00	486.94	511.42	479.66
MT133	0.62	0.62	508.75	485.19	468.18	477.61
MT92	0.62	0.62	497.50	497.45	491.96	478.00
MT112	0.61	0.61	455.00	469.96	468.30	495.96
MT118	0.58	0.59	500.00	487.11	502.14	474.97
MT139	0.61	0.61	482.50	462.54	468.56	482.77
MT116	0.59	0.63	470.08	471.74	475.81	488.85
MT135	0.60	0.62	510.00	485.89	478.73	463.21
MT115	0.60	0.59	475.00	463.80	470.09	484.11
MT80	0.58	0.61	518.75	488.53	500.10	479.07
MT128	0.58	0.59	487.50	463.08	485.98	503.41
MT129	0.58	0.59	497.50	506.60	482.93	490.67

*Note:* The average *R*_*f*_ value when TLC was run using standard metformin was 0.604. All data are reported as the average of triplicate measurements. Values in italics highlight deviations from the acceptable range.

## Data Availability

Data have been presented in the manuscript and supporting information.

## Supporting Information

Contains a Table reporting repeatability of *TLC Analyzer* in terms of concentration and comparison with the ImageJ method, and a representative HPLC chromatogram to show peak detection.
