# Supplementary material for: Smartphone-Assisted Thin-Layer Chromatography for Rapid Quality Screening of Metformin
Source: Adv Pharmacol Pharm Sci. 2025 Jun 18;2025:3306550. doi: 10.1155/adpp/3306550 (PMC12197440; doi:10.1155/adpp/3306550)
Supplement: Supporting Information — Additional supporting information can be found online in the Supporting Information section. [file 3306550.f1.docx]

**Smartphone-Assisted Thin Layer Chromatography for Rapid Quality Screening of Metformin**

Ram Kumar Bhattarai^1^, Sanam Pudasaini^1^, Toni Barstis^2^ and Basant Giri^1^

^1^Center for Analytical Sciences, Kathmandu Institute of Applied Sciences, Kathmandu, Nepal

^2^Department of Chemistry and Physics, Saint Mary’s College, Notre Dame, Indiana, USA

**Correspondence:**

E-mail: bgiri@kias.org.np

PO Box: 23002, Kathmandu, Nepal

**Supplementary information**

Table S1: Repeatability of TLC Analyzer in terms of concentration and comparison with ImageJ method

|  | Metformin (mg/mL) | | |
| --- | --- | --- | --- |
|  | 4 | 2 | 1 |
|  | 3.96 | 2.10 | 0.98 |
|  | 3.96 | 2.18 | 1.03 |
|  | 4.02 | 2.03 | 1.00 |
| ImageJ | 3.99 | 1.98 | 0.93 |
|  | 3.97 | 2.01 | 1.04 |
|  | 3.98 | 1.99 | 0.92 |
| Average | 3.98 | 2.05 | 0.98 |
| Stdev | 0.02 | 0.07 | 0.05 |
| %CV | 0.5 | 3.5 | 4.8 |
|  |  |  |  |
|  |  |  |  |
|  | 3.97 | 2.01 | 0.98 |
|  | 4.02 | 1.97 | 1.03 |
| TLC Analyzer | 4.07 | 1.96 | 1.12 |
|  | 3.89 | 2.12 | 0.97 |
|  | 4.09 | 1.97 | 0.96 |
|  | 3.98 | 2.11 | 1.1 |
| Average | 4.00 | 2.02 | 1.03 |
| Stdev | 0.07 | 0.07 | 0.07 |
| %CV | 1.8 | 3.6 | 6.7 |

**Sample HPLC Chromatograms**

**Figure S1:** HPLC Chromatogram of standard metformin.

**Figure S2:** HPLC Chromatogram of sample MT-30.
